# Photocatalytic cyclohexane oxidation and epoxidation using hedgehog particles

**DOI:** 10.1038/s41467-023-36473-5

**Published:** 2023-02-15

**Authors:** Douglas G. Montjoy, Elizabeth A. K. Wilson, Harrison Hou, Joel D. Graves, Nicholas A. Kotov

**Affiliations:** 1 Department of Chemical Engineering, University of Michigan, Ann Arbor, MI USA; 2 Biointerfaces Institute, University of Michigan, Ann Arbor, MI USA; 3 Department of Biomedical Engineering, University of Michigan, Ann Arbor, MI USA; 4 Department of Materials Science, University of Michigan, Ann Arbor, MI USA

**Keywords:** Chemical engineering, Structural properties, Photocatalysis

## Abstract

Inorganic particles are effective photocatalysts for the liquid-state production of organic precursors and monomers at ambient conditions. However, poor colloidal stability of inorganic micro- and nanoparticles in low-polarity solvents limits their utilization as heterogeneous catalysts and coating them with surfactants drastically reduces their catalytic activity. Here we show that effective photo-oxidation of liquid cyclohexane (CH) is possible using spiky particles from metal oxides with hierarchical structure combining micro- and nanoscale structural features engineered for enhanced dispersibility in CH. Nanoscale ZnO spikes are assembled radially on α-Fe_2_O_3_ microcube cores to produce complex ‘hedgehog’ particles (HPs). The ‘halo’ of stiff spikes reduces van der Waals attraction, preventing aggregation of the catalytic particles. Photocatalysis in Pickering emulsions formed by HPs with hydrogen peroxide provides a viable pathway to energy-efficient alkane oxidation in the liquid state. Additionally, HPs enable a direct chemical pathway from alkanes to epoxides at ambient conditions, specifically to cyclohexene oxide, indicating that the structure of HPs has a direct effect on the recombination of ion-radicals during the hydrocarbon oxidation. These findings demonstrate the potential of inorganic photocatalysts with complex architecture for ‘green’ catalysis.

## Abstract (web summary)

Spiky hedgehog particles are reported to access a photocatalytic pathway for alkane oxidation directly to industrially relevant epoxides in one step, providing a lower cost and less energy-intensive pathway as compared to common alkene feedstocks.

## Introduction

The partial oxidation of alkanes and alkenes represent some of the most common industrial reactions typically carried out in the gas phase at high temperature/pressure over a variety of inorganic catalysts. The realization of room temperature, liquid phase oxidation can potentially save vast amounts of energy and reduce greenhouse gas emissions. However, inorganic micro- and nanoparticle catalysts suffer from agglomeration in liquid hydrocarbons, which drastically reduces their catalytic activity^1–3^. Surfactants with long hydrocarbon ‘tails’ can aid in dispersing inorganic particles in hydrophobic media, but they also passivate the particle surface and reduce accessibility to active sites^4–6^. Dispersion agents of different types also compete with the alkanes and alkenes in the same oxidation reactions and reduce product selectivity as well as yield^7^. The implementation of particles with native surfactant-free inorganic surfaces allows for enhanced catalytic activity and reduced cost. Furthermore, such particles may also reveal new reaction pathways due to improved particle dispersion, wettability, and a different dielectric environment favoring unusual products^8^.

One of the examples of partial oxidation of hydrocarbons by O_2_ is the gas-phase oxidation of cyclohexane (CH). It leads to an unstable cyclohexyl hydroperoxide (CHHP) that decomposes in-situ into a mixture of cyclohexanone (CHone) and cyclohexanol (CHol) known as KA-oil^9^. This well-known chemical feedstock is produced at 8000 kilotons annually, primarily for Nylon^TM^ manufacturing^10^. The commercial process for KA-oil production involves the gas-phase oxidation of CH with O_2_ at high temperature (150 °C) and pressure (1-2 MPa). This high energy cost of this large-scale process is compounded by low chemical conversion of CH (4–15%) and inefficient heat recovery^10^. A wide array of alternative methods to synthesize Nylon^TM^ precursors including the photocatalytic oxidation of CH using titanium dioxide particles have been proposed^11,12^, but the poor dispersion of inorganic particles leads to a drastic reduction of catalytic efficiency and represents a fundamental barrier to improving the sustainability of plastic manufacturing.

A reliable pathway to enhance the dispersion of colloids without requiring chemical surface modification in nonpolar media is through decoration with stiff nanoscale spikes that markedly reduce van der Waals forces between particles, producing ‘hedgehog’ particles (HPs)^13^. The spiky geometry of HPs is inspired by the multitude of similarly shaped complex nano-, micro-, and submillimeter scale particles known in biology: viruses, algae, pollen. In all of these and other cases, spiky bio-particles utilize complex multiscale architecture to control agglomeration and interactions with other particles and surfaces. Complex inorganic HPs with ZnO spikes have also been shown to exhibit unusually high colloidal stability in ‘unfriendly’ media^13^. HPs showcase improved photocatalytic activity due to the enhanced electric field at the spike-media interface^8^. The unusual colloidal and chemical properties of HPs enable the direct access of organic molecules to the surface of the nanoscale spikes, opening the door to HP utilization in heterogeneous hydrocarbon photo-oxidation.

In this study, we address the problem of high-energy consumption in the partial oxidation of CH by taking advantage of the dispersibility of HPs in hydrophobic media. Instead of gas phase O_2_, we use hydrogen peroxide (H_2_O_2_), a strong electron acceptor and “green” oxidant^14^. Photo-oxidation of CH was carried out with HPs in a Pickering emulsion based on the colloidal properties of HPs and the translational prospects of such emulsions for green catalysis^15–17^. The hydrophilic phase in the Pickering emulsions can conveniently accommodate high concentrations of H_2_O_2_, which is difficult for hydrophobic media. Unlike known pathways for the (photo)oxidation of cyclic hydrocarbons, we observed the formation of not only CHone and CHol but also cyclohexene oxide (CHO). The observed epoxide is a valuable chemical intermediate used in a wide array of products including epoxy resins and elastomers, pesticides, stabilizers for halogenated hydrocarbons, and pharmaceuticals^18,19^.

## Results

### Catalyst design

**Fig. 1:**
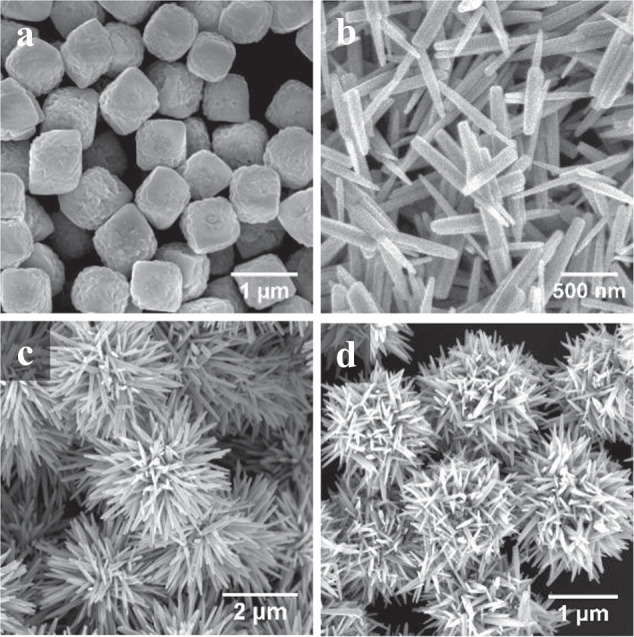
Hedgehog particle catalysts and component parts. SEM images of **a** hematite microcubes (Fe_2_O_3_ MCs), **b** ZnO nanorods (ZnO NR), **c** 3.7-ZnO/Fe_2_O_3_ hedgehog particles (HPs), and **d** 1.9-ZnO/Fe_2_O_3_ HPs.

ZnO is an attractive photo-oxidation catalyst for a variety of organic substrates due to its wide band gap and stability. ZnO has been extensively used in aqueous media where it has displayed high efficacy of photo-oxidation for environmental remediation^20–22^. One-dimensional nanoscale rods (nanorods, NRs) effectively delocalize charge carriers improving charge separation and, thus, the efficiency of redox reactions^21,23^. Additionally, nanostructures with high aspect ratios have been found to have enhanced photocatalytic activity due to increased amounts of surface defects^24,25^. With this context, we engineered HPs with ZnO spikes expecting that interfacial electron transport at an aqueous/nonpolar interface would lead to improvements in activity and selectivity while retaining the advantages of long ZnO nanostructures. HPs were synthesized on hematite microcube cores (Fe_2_O_3_ MCs) due to their high chemical stability and potential photocatalytic contributions due to redox activity of Fe^3+^ (Fig. 1a). Briefly, ZnO nanoparticles (NPs) were deposited on Fe_2_O_3_ MCs followed by sonothermal growth of ZnO NR to form spiky HPs of different sizes (3.7-ZnO/Fe_2_O_3_ HP and 1.9-ZnO/Fe_2_O_3_ HP shown in Fig. 1c, d, with particle dimensions listed in Table S1). To investigate the effect of the surface corrugation of particles on catalytic activity, individual ZnO NRs (Fig. 1b) and Fe_2_O_3_ MCs were also tested for catalytic activity in comparable conditions.

### Emulsion behavior

**Fig. 2:**
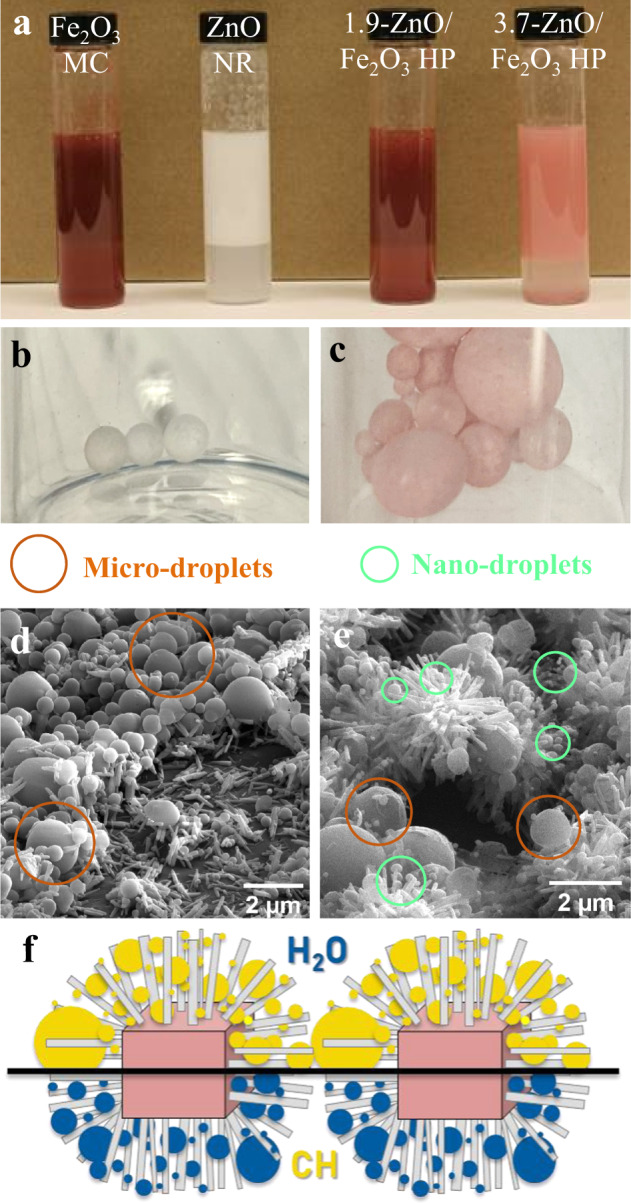
Emulsions of hedgehog particles and their component parts. Pickering emulsions formed after dispersing catalysts in a mixture of 1:1 (by vol) cyclohexane and water after 1 min **a**. Macroscale droplets formed during the polymerization of a 1:1 styrene/water emulsion for ZnO NR **b** and 3.7-ZnO/Fe_2_O_3_ HP **c**. Multiscale heterogeneity observed in SEM images of the surface of large macroscale polymer droplets showing micro- and nano-droplets interacting with ZnO NR **d** and 3.7-ZnO/Fe_2_O_3_ HP **e**. Graphic depicting behavior of dispersed HPs at the CH/H_2_O interface with micro- and nano-droplets **f**.

Inorganic particles can produce Pickering emulsions^15–17^ and take advantage of selective adsorption at the interface between immiscible fluids^26^ such as H_2_O_2_ and CH. The abundance of interfaces in emulsions can improve the chemical conversion of hydrocarbons^27^ that can be of further advantage when interfacial particles are photocatalytic^28^. We hypothesized that the unusual colloidal properties of HPs are favorable for the formation of Pickering emulsions (even without surfactant coatings on particles used to stabilize such emulsions^29^), while their spiky geometry retains particle dispersion and simplifies substrate access to the catalytic surface. HPs with short and long spikes, along with uncoated Fe_2_O_3_ MCs and ZnO NRs alone were investigated in a biphasic emulsion of CH and water. All were found to effectively stabilize a Pickering emulsion between the aqueous phase and CH upon mixing (Fig. 2a).

To evaluate the microscopic organization of particles in HP- and NR-based Pickering emulsions, an emulsion was made with styrene and water and subsequently polymerized to create particle-bound solid polystyrene droplets (Fig. 2b, c). The macro-droplets in Fig. 2b, c were then imaged using SEM (Fig. 2d, e). The images revealed that individual ZnO NRs, not incorporated into HP structures, primarily lay parallel to the surface of the macro-droplet, with many micro-droplets aggregated (Fig. 2d). On the contrary, HPs trap micro-droplets and even smaller nano-droplets on the surface of spikes and between spikes (Fig. 2e) providing extensive contacts to facilitate photocatalytic reactions. Superior wetting of HPs with the organic phase compared to NRs can also be identified analyzing the SEM images of a broken emulsion (Fig. S1). HPs are unique in their ability to stabilize the emulsion, remaining at the liquid–liquid boundary for extended time (Fig. 2f). Traditional hydrophilic catalysts such as the non-functionalized core microparticles and nanorods are rehydrated quickly, causing dispersion in the bulk aqueous phase where the photocatalytic oxidation of the organic phase is not as effective^28^. In addition to providing holes for oxidation, the halo of spikes should increase mass transport between the phases and to the ZnO surface by increasing the Pickering emulsion stability^30^.

### Factors influencing reaction pathway

**Fig. 3:**
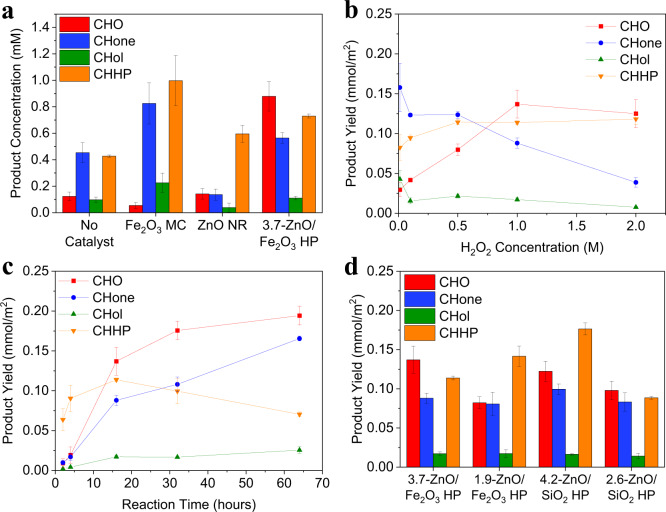
Product yield as a function of catalyst, H_2_O_2_ concentration, reaction time, and material. Product distributions from the photo-oxidation of CH in a 1:1 (by vol) CH/aqueous H_2_O_2_ emulsion with 1 mg/mL catalyst under broad spectrum light **a** with added 1 M H_2_O_2_ for 16 h, **b** as a function of added H_2_O_2_ concentration with 3.7-ZnO/Fe_2_O_3_ HP for 16 h, **c** as a function of reaction time with 3.7-ZnO/Fe_2_O_3_ HP and 1 M H_2_O_2_, and **d** HPs of different support materials and spike lengths with added 1 M H_2_O_2_. Data normalized by particle surface area except in **a** where the control reaction cannot be normalized. Error bars are a standard deviation calculated from at least three replicate runs. Source data are provided in a supplementary source data file.

With a significant difference in the colloidal properties and structure of the catalytic particles, one may expect to see not only changes in reaction efficiency but also in the product distribution. HPs, along with their ‘building blocks’ of Fe_2_O_3_ MCs and ZnO NRs, were investigated for CH oxidation in a biphasic system with 1 M H_2_O_2_ to promote production of the expected intermediate alkyl hydroperoxide (Fig. 3a). H_2_O_2_ is degraded by light without the presence of a catalyst, producing active ·OH capable of CH oxidation to CHHP and CHone, and small amounts of CHol^9^. CHO is also identified as a minor reaction product, indicating that one-pot liquid alkane epoxidation is possible in this solution environment. CHHP and CHone are produced with Fe_2_O_3_ MCs, likely from a Fenton-type mechanism with hydrogen peroxide^31–33^. With 3.7-ZnO/Fe_2_O_3_ HP, CHO emerges as the primary product, a remarkable finding given that the direct epoxidation of alkanes is reportedly problematic in traditional gas phase and even liquid phase conditions^34^. When this data is normalized by catalyst surface area (Table S2, Fig. S2), Fe_2_O_3_ MC activity is greater than that of ZnO NR and 3.7-ZnO/Fe_2_O_3_ HP, however, only a trace amount of CHO is produced. To our knowledge, the formation of the epoxide from CH in a single reaction has not yet been reported in literature.

The concentration of hydrogen peroxide was varied to further understand its role in the reaction mechanism. A progressive increase in CHO is seen as the concentration of added hydrogen peroxide is increased to 1 M, after which increasing the oxidant concentration does not benefit product formation (Fig. 3b, S2). The decrease in CHone and CHol production indicates that epoxidation is favored over the production of KA oil with increasing water content (Fig. S3) and oxidant concentrations (Fig. 3b, S2) in solution or adsorbed on the catalyst surface^3,35^. A lower H_2_O_2_ concentration of 12 mM consistent with studies of Fenton mechanisms was also explored (Fig. S2). In this case, CHone remains the primary product for all catalyst variations, highlighting the role of the solution environment on product selectivity. A kinetic study of products over time for the 3.7-ZnO/Fe_2_O_3_ HPs was performed to gain better insight into potential intermediates (Fig. 3c, S2). CHO yield increases to over 1.2 mM after 64 h. Corresponding with a slower rate of CHO production is a significant decrease in the concentration of CHHP from 16 to 64 h in indicating it is an intermediate for epoxidation^3,35,36^. In addition, CHone yield increases by a factor of close to two times over 64 hours, indicating that KA oil production may compete with epoxidation for either active sites or intermediate species such as CHHP^3^.

To determine the contributions of ZnO and the Fe_2_O_3_ MC support, HPs with silica (SiO_2_) spherical cores of different diameters (4.2-ZnO/SiO_2_ HPs and 2.6-ZnO/SiO_2_ HPs, Fig. S4) were compared to different size Fe_2_O_3_-supported HPs to determine the influence of an inert core material on the reaction products. When normalized by particle surface area (Fig. 3d, data without normalization are given in Fig. S2), HP catalysts of similar sizes are similarly productive regardless of core material, indicating that the core has minimal catalytic activity and that ZnO is the active catalyst for ketone and epoxide formation. A similar concentration of CHHP is produced with the SiO_2_-supported HPs, confirming that the Fenton mechanism seen with Fe_2_O_3_ MCs alone is negligible within HP heterostructure. When normalized by ZnO mass fraction (Fig. S5), however, the 4.2-ZnO/SiO_2_ HP falls below the 2.6-ZnO/SiO_2_ HP and the rest of the Fe_2_O_3_-containing heterostructured materials. This may be due to a lower ZnO aspect ratio^25^ or due to electronic transfers between the Fe_2_O_3_ MC in contact with ZnO (see SI for discussion). The argument that the spiky geometry of the particles is the main contributor in the selectivity of the ZnO surface remains valid as evidenced by comparing the results of Fig. 3d to ZnO NR in Fig. 3a.

### Engineering of HPs for photocatalytic reactions

**Fig. 4:**
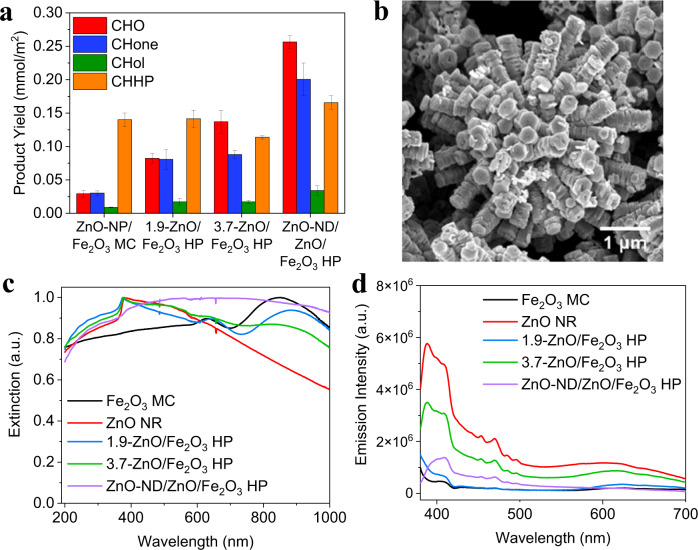
Understanding the influence of HP morphology on reaction. **a** Product yield from the oxidation of CH in a 1:1 (by vol) CH/aqueous H_2_O_2_ emulsion with 1 mg/mL catalyst and 1 M H_2_O_2_ under broad spectrum light for 16 hours for various ZnO-containing catalysts, normalized by particle surface area. **b** SEM of ZnO-ND/ZnO/Fe_2_O_3_ HP consisting of two-dimensional ZnO nanodiscs assembled around existing ZnO spikes. **c** Normalized UV–vis spectroscopy and **d** photoluminescence emission spectra of various ZnO-containing catalysts normalized by ZnO mass content, except for Fe_2_O_3_ MC that has no ZnO content. Error bars are a standard deviation calculated from at least three replicate runs. Source data are provided in a supplementary source data file.

The versatility of HP geometry, size, and material composition while retaining dispersibility allows for detailed studies of how particle morphology impacts photocatalytic activity. The influence of the ZnO nanostructure in HP catalysts on the product distribution, and in particular, the formation of CHO was investigated (Fig. 4a, S6). ZnO nanoparticles deposited on Fe_2_O_3_ MC (ZnO-NP/Fe_2_O_3_ MC, Fig. S6) were compared to HPs with zinc oxide spikes of various dimensions. In addition, 3.7-ZnO/Fe_2_O_3_ HPs functionalized with 2D concentric ZnO nanodiscs (ZnO-ND/ZnO/Fe_2_O_3_ HP; Fig. 4b) were tested to further understand the effect of nanostructure. The product yield and selectivity for ZnO-NP/Fe_2_O_3_ MC is like that of a 1:1 by mass heterogeneous mixture of Fe_2_O_3_ MCs and ZnO NRs (Fe_2_O_3_ MC + ZnO NR; Fig. S6—also includes 1:2 and 2:1 ratios) but neither match the activity of the Fe_2_O_3_ MC or 3.7-ZnO/Fe_2_O_3_ HPs and even hinders the reaction compared to un-catalyzed CHone production (Fig. 3a). With the introduction of ZnO in the system, CHone production greatly decreases, and CHO emerges as an abundant product, although high product yield is not seen without strong surface corrugation. The decrease in CHone likely can be attributed to two factors, one being the absorption of light by excess H_2_O_2_ which has already been determined to favor the epoxidation pathway and another being that the presence of ZnO limits the photo-Fenton reaction of the Fe_2_O_3_ MC.

From the trend in Fig. 4a, it is apparent that both ZnO and the core-spike HP structure is critical for selective epoxidation to occur, compared to minimal CHO produced on ZnO NRs (Fig. 3a). ZnO NRs have a higher surface area (7.4 m^2^/g) than the 3.7-ZnO/Fe_2_O_3_ HP (6.4 m^2^/g) and smaller dimensions than the spikes of 3.7-ZnO/Fe_2_O_3_ HP (109 vs. 120 nm width and 771 vs. 1530 nm length, respectively, listed in Table S1), which could be responsible for lower catalyst efficiency^25^. If smaller ZnO dimensions were hindering reaction productivity, one would expect the 1.9-ZnO/Fe_2_O_3_ HP (Figs. 1d and 4a, with smaller spike width and length dimensions of 78 and 588 nm, respectively) to perform poorly compared to the ZnO NR control. Instead, 1.9-ZnO/Fe_2_O_3_ HP outperforms the ZnO NRs by at least 2×, confirming that the structure of HPs assists in improving the photocatalytic activity of ZnO.

Photocatalytic activity also increases as the dimensions of HP spikes increase (from left to right across Fig. 4a). The increased product yield with ZnO-ND/ZnO/Fe_2_O_3_ HP (even with its low 4.4 m^2^/g surface area) is consistent with other studies of nanodiscs showing that enhancing populations of certain facets along the disc translate to higher catalytic activity^37,38^. However, XRD of the ZnO-ND/ZnO/Fe_2_O_3_ HPs shows similar crystal structure to the ZnO/Fe_2_O_3_ HPs (Fig. S7), indicating that the enhancement likely stems from the macro-scale assembly of the particle structure rather than the crystallization process during synthesis. In Fig. S8, epoxide selectivity was calculated for HPs of various lengths in comparison to other metal oxide photocatalysts and controls including Fe_2_O_3_ MCs, ZnO NRs, Fe_2_O_3_ MC + ZnO NR, ZnO-NP/Fe_2_O_3_ MCs, 1.9-ZnO/Fe_2_O_3_ HP, 3.7-ZnO/Fe_2_O_3_ HP, and ZnO-ND/ZnO/Fe_2_O_3_ HP. ZnO-ND/ZnO/Fe_2_O_3_ HP and 3.7-ZnO/Fe_2_O_3_ HP showed the highest selectivities to epoxide of 39% and 38% respectively while non-spiky catalysts such as the ZnO-NP/Fe_2_O_3_ MCs showed lower selectivities of 10–15%.

Many HPs exhibit broadband scattering in UV–vis spectra (Fig. 4c) as described in previous studies^8,13,39,40^. The optimal concentration for the 3.7-ZnO/Fe_2_O_3_ HP catalyst was found to be 0.5 mg/mL with a decrease in activity at high concentrations (Fig. S9), likely due to scattering. Increased activity of HPs and ZnO-ND/ZnO/Fe_2_O_3_ HPs can partially be explained by improved electron–hole separation in the ZnO spikes. We found that HP catalysts have lower photoluminescence (PL) emission intensity (Fig. 4d, S10) than ZnO NRs and a mixture of Fe_2_O_3_ MC + ZnO NR when normalized by the mass fraction of ZnO in the catalyst, signifying less electron–hole recombination^41^.

### Cyclohexane dehydrogenation and confirmation of epoxidation mechanism

ZnO has been reported as an active catalyst for propene photo-epoxidation^42^, but the photocatalytic oxidative dehydrogenation (ODH) necessary for CHene formation has only been reported in gas phase with oxygen as an oxidant^43,44^, and has not been reported in the liquid phase at ambient conditions to our knowledge. Small concentrations of CHene were not easily detectable in our system, but for select reactions where it was quantified, CHene production is higher at 1 M H_2_O_2_ than at 12 mM, supporting the hypothesis that CHene is the second intermediate in CHO formation (Table S3). The addition of 1 mM CHene at the beginning of the reaction (Fig. S11) slightly inhibits the reaction, lowering the production of CHHP and CHO. If cyclohexene is indeed the second intermediate, we believe the presence of high levels of CHHP at the start of the reaction are required for the epoxidation reaction to occur (Fig. 3a, c) CHol was ruled out as an intermediate for both CHene and CHone formation (Fig. S11). With the lack of additional alkenyl species such as cyclohexen-2-one (negligible amounts detected), the hydroperoxidation of the allylic carbon atom via an H-abstraction by a HO• radical is not favored, while epoxidation is. The presence of water and the acidic aqueous environment are responsible for enhanced oxidation of CH to CHene^45,46^ and epoxidation of CHene^47,48^, assisted by the increased phase contact supported by the HP structure. However, in acidic aqueous solvents, epoxide stability is reportedly low due to hydrolytic ring cleavage^35,36^, especially in more miscible systems with co-solvents^49^. Utilizing CH as both solvent and substrate allows for epoxide accumulation and stability in the bulk nonpolar phase after desorption from the catalyst surface at the phase boundary.

**Fig. 5:**
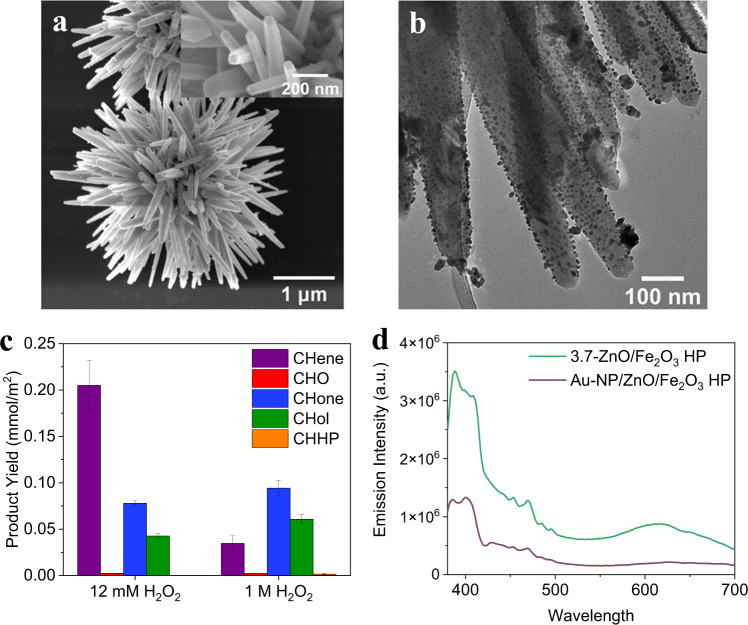
Cyclohexane dehydrogenation with the introduction of gold nanoparticles. SEM **a** and TEM **b** images of 3.7-ZnO/Fe_2_O_3_ HPs decorated with Au nanoparticles, denoted Au-NP/ZnO/Fe_2_O_3_ HP (average nanoparticle size: 12.9 ± 3.6 nm). **c** Product distribution normalized by particle surface area for 1 mg/mL Au-NP/ZnO/Fe_2_O_3_ HP in the photo-oxidation of CH in a 1:1 (by vol) CH/aqueous H_2_O_2_ emulsion under broad spectrum light for 16 hours at low (12 mM) and high (1 M) H_2_O_2_ concentrations. **d** Photoluminescence emission spectra for Au-NP/ZnO/Fe_2_O_3_ HP normalized by ZnO mass content. Error bars are a standard deviation calculated from at least three replicate runs. Source data are provided in a supplementary source data file.

**Fig. 6:**
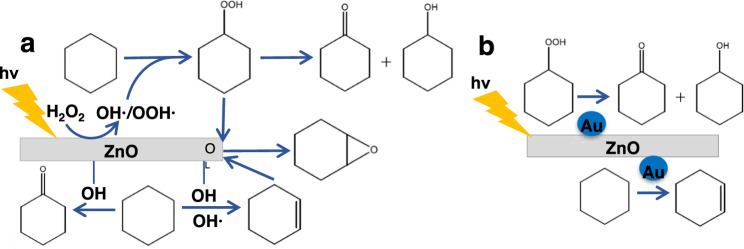
Cyclohexane oxidation, dehydrogenation, and epoxidation pathways. **a** Photocatalytic oxidation of cyclohexane with ZnO nanorod present in HP catalyst. ·OH/·OOH radicals are produced from excitation of H_2_O_2_ and ZnO which form cyclohexyl hydroperoxide. ZnO OH sites result in dehydration to cyclohexene which is then combined with cyclohexyl hydroxide at a lattice OH site (O_L_) to form cyclohexene oxide. **b** Au NPs (blue dots, not to scale) occupy OH lattice sites, efficiently resulting in dehydrogenation as well as degrading hydroperoxide species immediately, preventing epoxide formation. The reaction is dependent on water content of the reaction (Fig. S3) and can be further accelerated by increasing the temperature (Fig. S16).

The concentration of -OH groups on the surface has been shown to correlate with catalytic activity in CH oxidation. The addition of gold NPs (Au-NP/ZnO/Fe_2_O_3_ HP; imaged in Fig. 5a, b), which reduce available -OH groups on the ZnO surface^50^, results in an order of magnitude drop in CHO formation compared to 3.7-ZnO/Fe_2_O_3_ HP at the same conditions, indicating the importance of native ZnO -OH groups or lattice oxygen defects for epoxidation (Fig. 5c). Au-NP/ZnO/Fe_2_O_3_ HPs give a large increase in CHol selectivity and yield at both oxidant concentrations with significant CHone production, consistent with previous studies of gold catalysts in H_2_O_2_-assisted CH oxidation^51^. Gold NPs also improve photon efficiency in the material likely through enhanced visible light absorption (UV–vis found in Fig. S12), localized surface plasmon resonance, or electron trapping, supported by its low PL emission (Fig. 5d). Figure 6 shows the expected reaction pathways at high oxidant concentrations on ZnO HP catalysts and Au-NP/ZnO/Fe_2_O_3_ HP catalysts. At low oxidant concentrations, -OH and -OOH groups on the surface of the catalyst are responsible for direct oxidation to CHone, likely replenished by O_2_ in a Mars-van Krevelen mechanism^52,53^. At 1 M H_2_O_2_, the likely pathway to CHO is through the dehydrogenation of CH to CHene, followed by an epoxidation reaction with CHHP to form CHO (Fig. 6a)^35,36,44,49^. The lack of CHHP remaining at the end of the reaction time with Au-NP/ZnO/Fe_2_O_3_ HP catalysts indicates gold accelerates de-peroxidation of CHHP to KA oil before it can accumulate in the CH phase (Fig. 6b). Unique to the gold-containing catalyst, CHene is seen here as a major reaction product at both oxidant concentrations, which normally requires elevated temperatures. This evidence, combined with the lack of (1) large amounts of CHene seen without Au and (2) remaining CHHP in solution after reaction with Au, confirms that the mechanism producing CHO goes through a CHene intermediate likely bound to a ZnO active site covered by Au.

### Recyclability and recovery of HP catalysts

We also evaluated the possibility of recovering HPs from dispersions by analyzing changes in chemical and physical structure to further understand robustness of the oxidation process (Fig. S13). Original and recovered catalyst show similar product yields. XPS and SEM were also used to analyze reaction site and catalyst morphology after reaction (Fig. S14). photoetching of the ZnO spikes in the presence of H_2_O_2_ is seen in SEM images which could reduce epoxide yield over time. In addition, O1s XPS spectrum shows a relative increase of O_D_ (oxygen near oxygen deficient regions or surface-bound peroxides) and decrease of O_L_ (O^2-^ lattice oxygen) assigned to Zn-O^54–57^, consistent with the degradation of the ZnO structure and peroxidation of the surface to ZnO_2_. This finding further supports that a lattice -OH site is responsible for epoxidation (Fig. 6).

## Discussion

HPs selectively oxidize CH directly into CHO in Pickering emulsions with H_2_O_2_. The HP structure facilitates formation of micro- and nano-droplets providing extensive contact area between water and CH, which is crucial for increased transport of reactants and CHO production. Through an extensive investigation of the catalyst composition and structure, the oxidant concentration and solution environment, we have found that direct CHO production involves simultaneous photocatalytic dehydrogenation and epoxidation on oxygen-containing surface groups on ZnO.

Note that CHO is industrially made from benzene via the epoxidation of a CHene^44,58^ using peracid catalysts which can have low reaction rates and acidic byproducts or with heterogeneous catalysts such as Ag^59–61^. The direct oxidation of CH to CHO has not yet been reported, likely due to the multistage reaction mechanism requiring both dehydrogenation and epoxidation, which is difficult to realize on standard heterogeneous or homogeneous catalysts^62–65^. Oxidative dehydrogenation of CH to CHene requires 200 °C and O_2_ as an oxidant (without oxygen, temperatures up to 500 °C are required)^66^. Photocatalytic oxidative dehydrogenation oxidation of CH was reported with sulphated MoO_x_ but in the gas phase at 120 °C^43^. We also note that the high temperatures required for dehydrogenation are not compatible with green oxidants such as H_2_O_2_ and room temperature epoxidation catalysts. From a practical perspective, the use of alkanes instead of alkenes as a feedstock for CHO offers a remarkably lower cost as well as reduced environmental impact. For example, the production of propylene from steam cracking results in 1200 kg CO_2_/g propylene compared to ca 230 kg CO_2_/g propane for production of propane from natural gas^67^. Catalysis with Pickering emulsions enabled by spiky particles has a direct impact on the broader field of liquid-phase chemical conversions of alkanes and organic substrates.

The exploration of HPs as a catalyst for epoxide formation from alkanes rather than olefins also has large implications. Ethylene oxide, propylene oxide and other industrially important epoxides would benefit largely in terms of economic and environmental cost from using alkane feedstocks rather than olefins^31^. This epoxidation pathway could lead to more efficient production of valuable chemical intermediates. Future work will focus on further control of selectivity through materials, oxidants and solvent environment and expansion to other alkane feedstocks.

## Methods

### Catalyst synthesis

Hematite core particles (Fe_2_O_3_ MCs) were synthesized using a literature hydrothermal method, shown in Fig. 1a^64^. Briefly, FeCl_3_·6 H_2_O and NaOH were dissolved into a mixture of distilled water and ethanol with a volume ratio of 2:1 and stirred for 2 h. They were then transferred to a Teflon-lined autoclave and reacted at 160 °C for 24 h. The precipitates were then centrifuged at 6000 RPM for 10 min and washed with ethanol one time and then water two times to yield a colloidal solution of hematite microcubes. The Fe_2_O_3_ MC were confirmed to be hematite using energy dispersive x-ray spectroscopy (EDX) and powder x-ray diffraction (XRD). Both ZnO-NP/Fe_2_O_3_ MC and ZnO/Fe_2_O_3_ HP were synthesized using an adapted method^13^ as described below. ZnO-NP/Fe_2_O_3_ MC were produced using a layer-by-layer film of negatively-charged poly(sodium 4-styrenesulfonate) (PSS) to conformally deposit positively-charged ZnO nanoparticle seeds onto Fe_2_O_3_ MCs. Briefly, 5 mL core was mixed with 25 mL PSS solution (1 mg/mL, 1 M NaCl) and incubated for 20 min. Cores were then centrifuged for 4000 RPM for 10 min and washed three times with water^8^. Fe_2_O_3_ HPs were synthesized using a scaled sonothermal method similar to previous studies^8,13^ but with a different starting core material. 1.25 mL of coated hematite solutions (2.5 wt%) was mixed with 40 mL ZnO seed solution (0.025 wt%) and incubated for 1 h^8,13^. Particles were then filtered using a track-etch membrane and combined with equimolar (37.5 mM) solutions of zinc nitrate hexahydrate (ZnH) and hexamethylenetetraamine (HMT) in water. The ZnO NPs on the Fe_2_O_3_ MCs were then sonothermally (Hielscher 1000UIP HdT sonicator for 90 min) grown into ZnO spikes, producing 1.9 µm diameter HP, denoted as 1.9-ZnO/Fe_2_O_3_ HP. 3.7 µm diameter HP, denoted as 3.7-ZnO/Fe_2_O_3_ HP were created by doing an additional sonication (90 min) with ZnH and HMT precursor solutions after purification. HPs were purified by removing excess nanorods after sedimentation of HPs. Excess ZnO spikes (ZnO NR) produced during HP synthesis were collected for photocatalytic testing, as shown in Fig. 1b. As a control to test the influence of the catalytic support, SiO_2_ HPs were also synthesized using the same method on non-catalytic silica spheres^65^ and are discussed in a previous study^8^. Additional details on synthesis of ZnO-ND/ZnO/Fe_2_O_3_ HP and Au-NP/ZnO/Fe_2_O_3_ HP are included in the supplementary information. All materials in this work were calcined in stagnant air at 500 °C for 1 h prior to photocatalytic screening unless otherwise noted.

### Emulsion studies

Emulsion characterization was performed via both visual confirmation (Fig. 2a) and by using polymerization of styrene-in-water emulsions to capture particle behavior at the organic/aqueous interface (Fig. 2b–e). For the visual emulsion confirmation, 30 mg of catalyst material was dispersed in 15 mL of ultrapure water and sonicated for 2 min. Then, 15 mL of CH was added, and the vial was shaken vigorously to stimulate the emulsion. The styrene polymerization procedure was taken directly from literature^66^. In brief, 4 mg of particles was dispersed in 2 mL of water and was sonicated for 20–30 min. A total of 10 µL of hydroquinone was added to the aqueous solution, which was then was mixed with 2 mL of styrene containing 37 mM 2,2′-Azobis(2-methylpropionitrile) (AIBN) as an initiator. After generating the emulsion, the samples were heated to 80 °C for 24 h to polymerize the styrene droplets.

### Electron microscopy

A total of 10 µL of sample was dropped onto a silicon wafer and allowed to evaporate, leaving dried particles. Samples were imaged using a FEI Nova 200 Nanolab SEM and a FEI Helios 650 Nanolab SEM/FIB.

### Optical spectroscopy

UV–vis spectroscopy data were collected on an Agilent Cary 8454 UV–vis spectrophotometer. Photoluminescence (PL) spectroscopy data was collected on a Horiba FluoroMax-3 monochromator with excitation of 360 nm. Samples were dispersed at 0.25 mg/mL water for both UV–vis and PL spectroscopy. HP dimensions in Table S1 were used to calculate mass fraction of ZnO in each sample assuming 200 spikes/particle for the normalization of PL data.

### Biphasic photocatalytic oxidation of cyclohexane

All reagents are from Millipore Sigma and were used as received. The catalyst was first dispersed in 15 mL CH via bath sonication for 15 s before being added to a 50 mL quartz round-bottom reaction flask. Next, ultrapure water and concentrated aqueous H_2_O_2_ (34.5–36.5%) were added to achieve a total aqueous volume of 15 mL and the desired H_2_O_2_ concentration. The catalyst and oxidant concentrations were calculated using the total volume of 30 mL which was consistent for every experiment in this report. The emulsion was incubated in the dark under vigorous stirring for 30 min prior to illumination.

A broad-spectrum X-Cite 120W light source with a radiant flux of 0.065 W/cm^2^ in our system was used for all experiments. Light intensity as a function of wavelength can be found in Fig. S15. Experiments showing the impact of light vs. heat can be shown in Fig. S16. Initial products were identified using a Shimadzu GC-MS (Fig. S17 shows confirmation of cyclohexene oxide with HP catalyst) and were quantified using the following procedure. After reaction, the sample was transferred to a scintillation vial. After 10 min to allow the phases to separate, two 1 mL aliquots from the cyclohexane phase was removed. To one aliquot, excess triphenylphosphine was added to convert all remaining cyclohexyl hydroperoxide to cyclohexanol based on a literature method^68^. After 15 min both of these were analyzed with a Gas Chromatograph (GC-2014A, Shimadzu) with a capillary flame ionization detector (FID) with an RTX-5 60m 0.5 µm column. An injector temperature of 270 °C was used and temperature was ramped 10 °C/min from 60 °C (hold for 1 min) to 260 °C (hold for 2 minutes). For each product, calibration standards were prepared and run to create a linear calibration curve used to quantify the concentration of each product in the sample.

For most experiments, the GC method did not allow for the quantification of CHene; Table S3 shows the CHene amounts quantified for specific single experiments where the GC method was changed to make CHene quantification possible. We also recognize that CO_2_ is formed in the gas phase from the complete oxidation of CH and its oxidation products but was not analyzed in most experiments due to system limitations. Each experiment reported was run in triplicate unless otherwise noted.

## Supplementary information

Supplementary Information

## Data Availability

The source data generated in this study for Figs. 3, 4, and 5 is available in the supplementary source data file uploaded. Source data are provided with this paper.

## Source data

Source Data
