# Supplementary material for: Photocatalytic cyclohexane oxidation and epoxidation using hedgehog particles
Source: Nat Commun. 2023 Feb 15;14:857. doi: 10.1038/s41467-023-36473-5 (PMC9932155; doi:10.1038/s41467-023-36473-5)
Supplement: Supplementary file 1 — Supplementary Information [file 41467_2023_36473_MOESM1_ESM.pdf]

Supporting Information

Cyclohexane oxidation and epoxidation using photocatalytic hedgehog particles

Douglas G. Montjoy[1,2,‡], Elizabeth A. K. Wilson[1,2,‡], Harrison Hou[1,2], Joel D. Graves[1], Nicholas A. Kotov[1,2,3,4]

[1]Department of Chemical Engineering, University of Michigan, Ann Arbor, Michigan 48109, USA
[2]Biointerfaces Institute, University of Michigan, Ann Arbor, Michigan 48109, USA
[3]Department of Biomedical Engineering, University of Michigan, Ann Arbor, Michigan 48109, USA
[4]Department of Material Science, University of Michigan, Ann Arbor, Michigan 48109, USA
‡These authors contributed equally
*Correspondence E-mail: kotov@umich.edu

Inventory of Supporting Information

Source Data for Figures 3,4, and 5 is included in a supplementary source data file (.xls)
Figure S1: Emulsion polymerization of styrene and Hedgehog Particles (HPs)
Table S1: Particle Dimensions of HP Catalysts and their components
Table S2: Surface area of catalysts determined using Brunauer-Emmett-Teller (BET) theory
Figure S2: Oxidation data from Figure 3 unnormalized or normalized by surface area.
Figure S3: Product yield as a function of the amount of water present in the reaction
Figure S4: SEM of ZnO/SiO_2 HP catalysts and UV-Vis extinction and photoluminescence spectra.
Figure S5: Product yield for select catalysts normalized by ZnO mass fraction.
Figure S6: Characterization of ZnO nanoparticle decorated Fe_2O_3 microcube and unnormalized data from Figure 4
Figure S7: X-ray diffraction of HP Catalysts including nanodisc functionalization and components.
Figure S8: Epoxide selectivity of catalysts in cyclohexane oxidation
Figure S9: Product concentration as a function of HP catalyst concentration
Figure S10: Photoluminescence emission spectra of microcube and nanorod mixture and non-normalized data from Figure 4d.
Table S3: Cyclohexene yield for select single experiments at low and high oxidant concentrations.
Figure S11: Product yield of HP catalyst with designated concentration of products added to reaction flask.
Figure S12: TEM images and UV-Vis extinction spectra of Au-NP/ZnO/Fe_2O_3 HP
Figure S13: Cyclohexane oxidation results with recycled HP Catalyst
Figure S14: XPS spectra and SEM of recycled HP catalyst
Figure S15: Light intensity for X-Cite Series 120 lamp used in cyclohexane oxidation experiments
Figure S16: Cyclohexane oxidation product distribution from various light and heat conditions
Figure S17: GC-MS mass spectrum identification of cyclohexene oxide in solution after reaction

Fig. S1: Emulsion polymerization of styrene with Hedeghog Particle and Nanorods

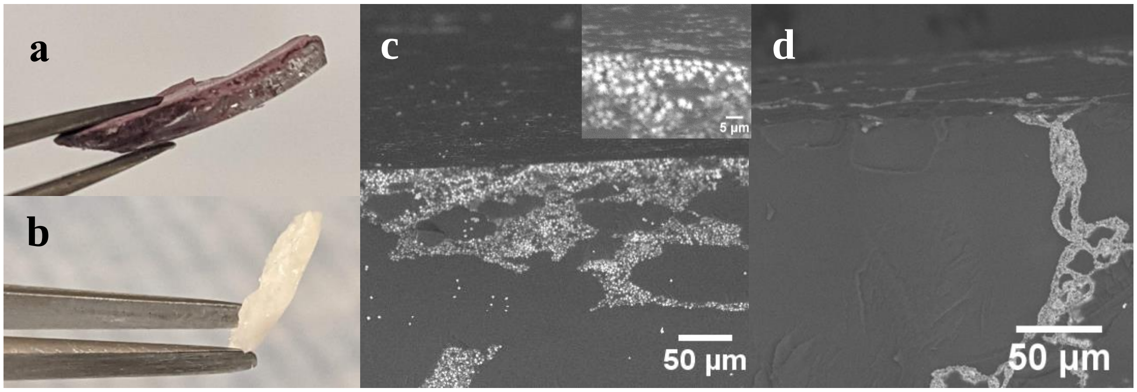

Fig. S1: Bulk phase organic polymerization of styrene containing 1 mg/mL (over both phases) 3.7-ZnO/Fe_2O_3 HP (**a**, **c**) and ZnO NR (**b**, **d**).

To visualize the interaction of particles with the bulk organic phase in the event that the emulsion broke, in one experiment, the emulsion was initiated and then agitated gently to break the organic droplets and polymerize the bulk organic phase. The results are shown in **Fig. S1**. In the bulk polymer, hedgehog particles (HPs) (**Fig. S1a, c**) show good dispersion within the organic layer while ZnO nanorods (NRs) self-assemble into aggregated chains (**Fig. S1b, d**), confirming that even when the emulsion is broken, HPs are engineered to retain dispersion and remain wetted by the organic phase and therefore retain higher available surface area for catalysis. SEM images were taken on a TESCAN RISE under low vacuum conditions without any conductive sputter coating.

Table S1: Particle Dimensions of HP Catalysts and their components

Catalyst	Spike Width (nm)	Spike Length (nm)	Total Diameter (nm)
Fe_2O_3 MC	N/A	N/A	689 ± 86 (edge length)
ZnO NR	109 ± 32	771 ± 242	N/A
1.9-ZnO/Fe_2O_3 HP	78 ± 20	588 ± 126	1865 ± 252
3.7-ZnO/Fe_2O_3 HP	120 ± 38	1530 ± 173	3748 ± 347
ZnO-ND/ZnO/Fe_2O_3 HP	338 ± 45	1820 ± 281	4328 ± 562
2.6-ZnO/SiO_2 HP[1]	72 ± 17	758 ± 56	2643 ± 113
4.2-ZnO/SiO_2 HP[1]	166 ± 27	1545 ± 240	4216 ± 480

Particle dimensions were determined by measuring features in SEM images, in which over 100 measurements were used for each dimension below. These dimensions were used to estimate the

mass fraction of ZnO per sample, assuming 200 spikes/particle and densities of 5.26 g/cm^3 for hematite, 5.61 g/cm^3 for zinc oxide, and 2.65 g/cm^3 for silica.

Table S2: Surface area of catalysts determined using Brunauer-Emmett-Teller (BET) theory

Catalyst	BET Surface Area (m^2/g)
Fe_2O_3 MC	1.849 ± 0.017
ZnO-NP/Fe_2O_3 MC	5.136 ± 0.064
ZnO NR	7.377 ± 0.045
1.9-ZnO/Fe_2O_3 HP	6.257 ± 0.022
3.7-ZnO/Fe_2O_3 HP	6.416 ± 0.046
ZnO-ND/ZnO/Fe_2O_3 HP	4.410 ± 0.007
2.6-ZnO/SiO_2 HP	8.227 ± 0.013
4.2-ZnO/SiO_2 HP	4.003 ± 0.010
Au-NP/ZnO/Fe_2O_3 HP	13.869 ± 0.028

Nitrogen physisorption data was collected on a Micromeritics ASAP 2020 Plus instrument. Samples were degassed under vacuum at 350°C for 6 hours prior to measurement. Eight points within the range of 0.1 to 0.3 P/P_0 were used to calculate the surface area of the materials. For most samples, a minimum of 5 m^2 in the analysis tube was used to ensure accuracy.

Figure S2: Oxidation data from Figure 3 unnormalized or normalized by surface area.

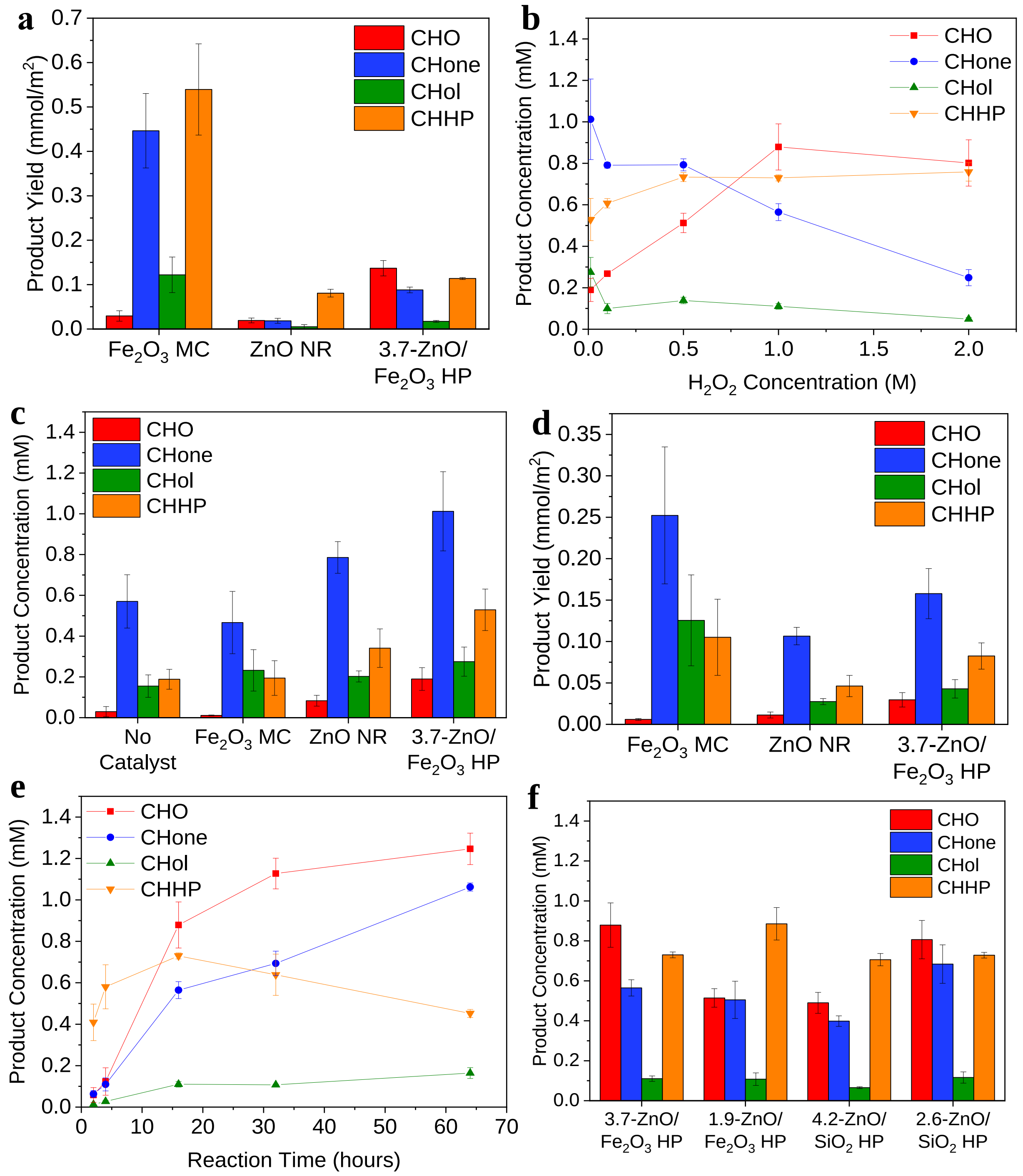

Fig. S2: Data from **Figure 3** unnormalized (**b**, **c**, **e**, **f**) or normalized by BET surface area (**a**, **d**). Reaction conditions: 1:1 (by vol) cyclohexane(CH)/aqueous H_2O_2 emulsion with 1 mg/mL catalyst under broad spectrum light for 16 hours (**a-d**, **f**) or designated reaction time (**e**) at designated H_2O_2 concentrations (**b**) or 12mm H_2O_2 (c, d) or 1 M H_2O_2 (**a**, **e**, **f**). High product yield with Fe_2O_3 MC (**a**, **d**) is due to its extremely low SA, but poor dispersion of Fe_2O_3 MC in nonpolar solvents limits the amount of catalyst that can be utilized. Error bars are a standard deviation calculated from at least three replicate runs.

Figure S3: Product yield as a function of the amount of water present in the reaction

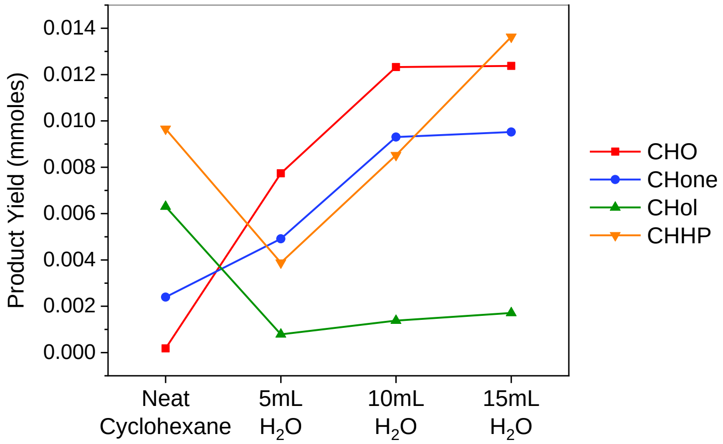

Fig. S3: Product yield as a function of the amount of water present in the reaction. The total reaction volume was kept constant at 30 mL with the ratio of water:CH increasing: 0:1 (neat cyclohexane with aqueous H_2O_2 at total concentration of 1M), 1:5 (5 mL H_2O), 1:2 (10 mL H_2O), 1:1 (15 mL H_2O). Experimental conditions: 16 hours, 1 M H_2O_2, 1 mg/mL 3.7-ZnO/Fe_2O_3 HP. Experiments all single runs except for 15 mL H_2O, which was a triplicate average.

Figure S4: SEM of ZnO/SiO_2 HP catalysts and UV-Vis extinction and photoluminescence spectra.

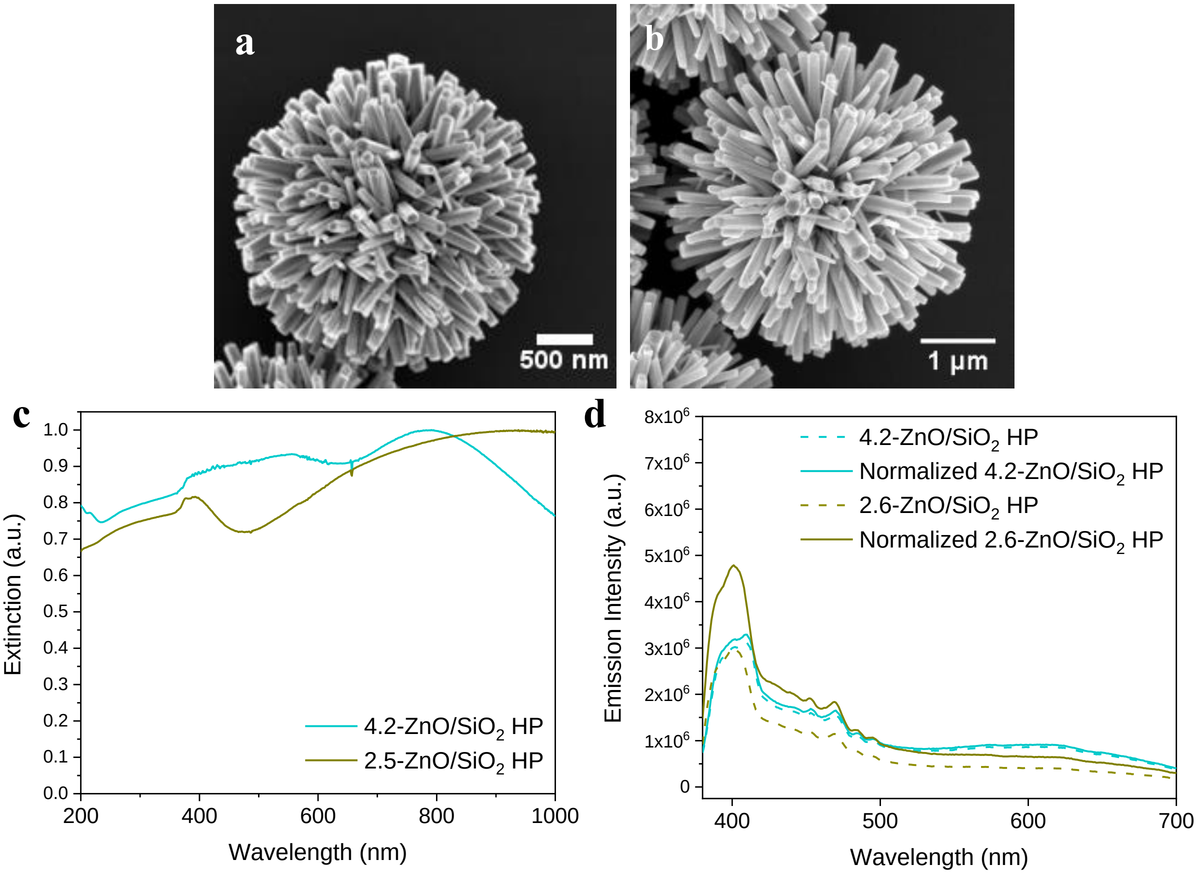

Fig. S4: SEM image of (**a**) 2.6-ZnO/SiO_2 HP and (**b**) 4.2-ZnO/SiO_2 HP catalysts for CH oxidation. Measurements are included in **Table S1**. (**c**) Normalized UV-Vis extinction spectra and (d) photoluminescence emission from excitation at 360 nm both with and without normalization by ZnO mass content.

Figure S5: Product yield for select catalysts normalized by ZnO mass fraction

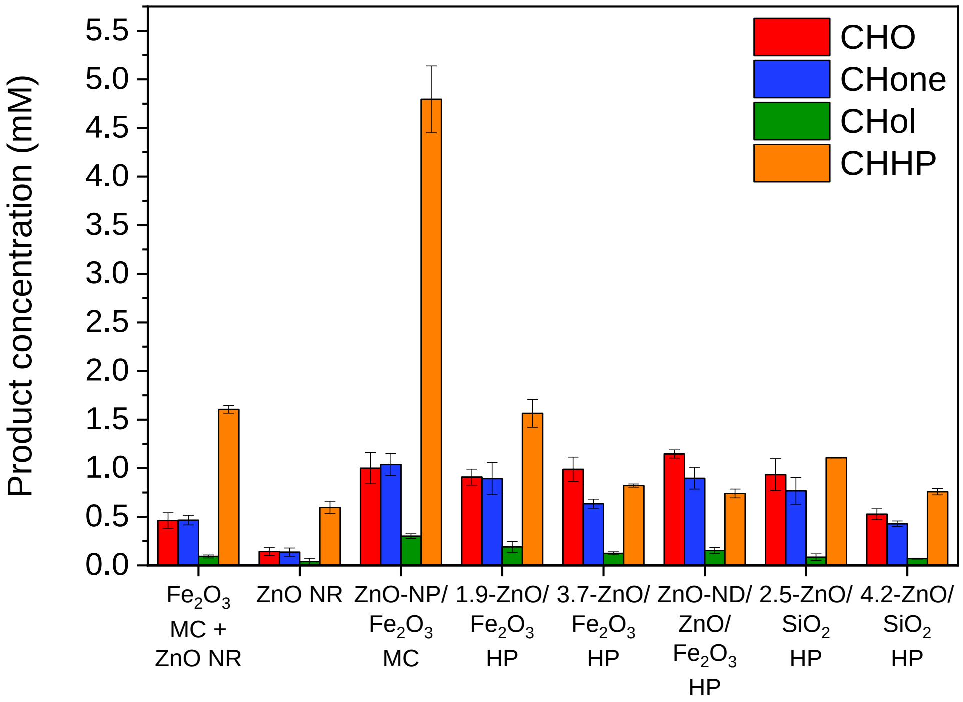

Fig. S5: Product yield for select catalysts normalized by ZnO mass fraction, calculated using dimensions of supports and spikes found in **Table S2**. Reaction conditions: 16 hours, 1M H_2O_2, 1 mg/mL catalyst, 1:1 by vol. mixture of cyclohexane and water. Error bars are a standard deviation calculated from at least three replicate runs.

We argue that the physical structure of the catalyst is the most important factor for the catalytic enhancements we have seen. However, we cannot completely rule out the possible electronic influence of the heterostructure of the HP catalyst.[1–7] The core material of a large HP structure likely does not absorb light due to light scattering within the particles,[8] so photo-induced transfers may be limited to those from ZnO to Fe_2O_3. Because of the influx of holes to the hematite core, electrons may be displaced toward ZnO. This theory is supported by the product yield and selectivity for various catalysts when normalized by the mass fraction of ZnO (**Fig. S5**). All catalysts where ZnO is bound to Fe_2O_3 show very similar amounts of epoxide and ketone yield, confirming ZnO as the only active catalyst when combined with another material. This may indicate an efficiency increase when Fe_2O_3 is used only as an electronic support material (and not able to absorb light itself) as epoxide and ketone formation is increased with all catalysts with contact between ZnO/Fe_2O_3 compared to separate Fe_2O_3 MC and ZnO NR including with a ZnO-NP/Fe_2O_3 MC. The ZnO-NP/Fe_2O_3 MC shows a high production of cyclohexyl hydroperoxide because this normalization strategy does not consider the hydroperoxide formation occurring anywhere other than ZnO. However, to disperse the same amount of ZnO as in HPs, over 15x higher concentration would be required for the ZnO-NP/Fe_2O_3 MC which would have poor dispersion and blocked light absorption due to the density of the slurry.

Figure S6: Characterization of ZnO nanoparticle decorated Fe_2O_3 microcube and unnormalized data from Figure 4

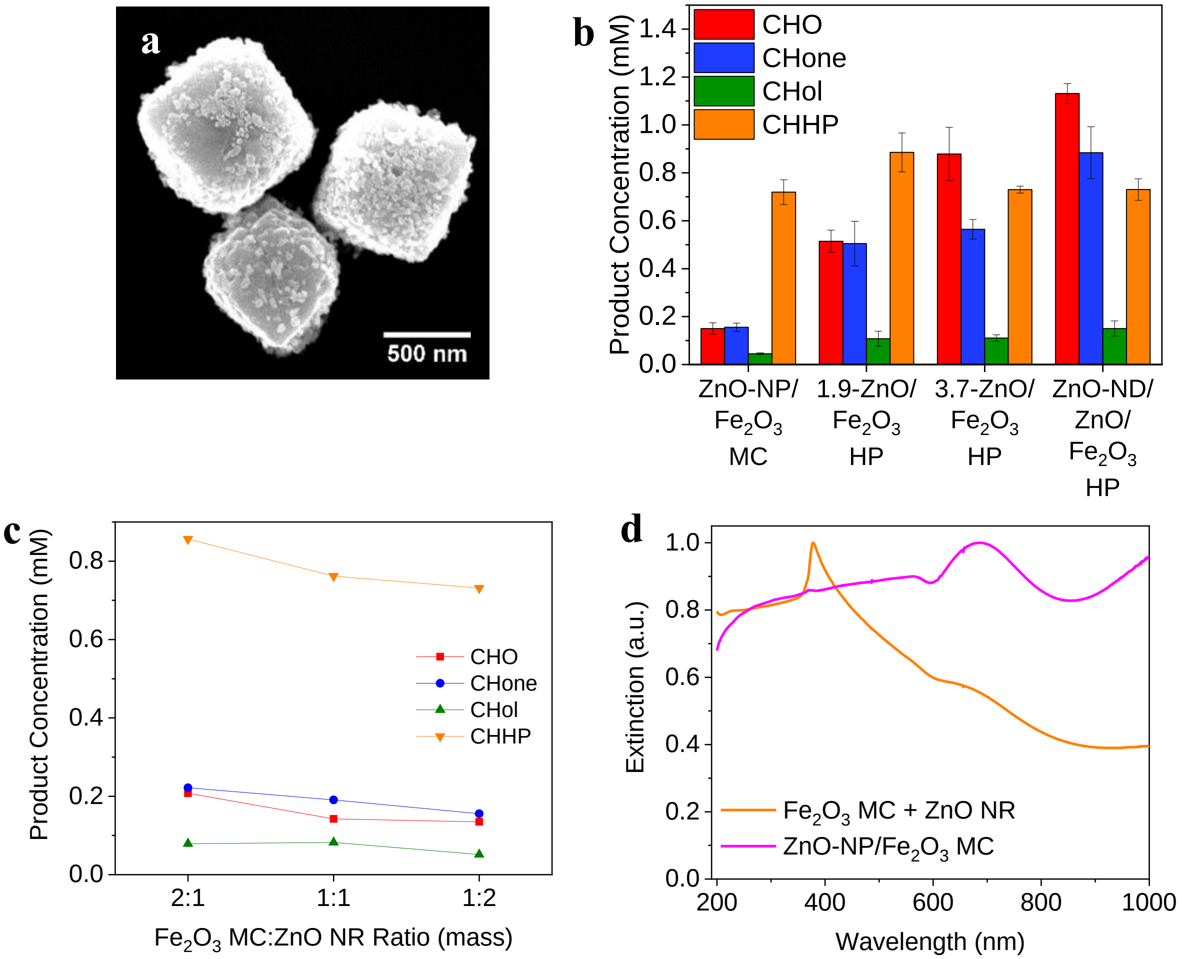

Fig. S6: (**a**) SEM image of ZnO NP decorated Fe_2O_3 MC (ZnO-NP/Fe_2O_3 MC), (**b**) data from **Figure 4** not normalized by BET surface area, (**c**) product distribution of CH photo-oxidation with various heterogeneous mixtures of Fe_2O_3 MC and ZnO NR (Fe_2O_3 MC + ZnO NR) given in Fe_2O_3 MC:ZnO NR ratio by mass (overall concentration of 1 mg/mL catalyst), and (**d**) UV-Vis extinction for ZnO-NP/Fe_2O_3 MC and a 1:1 Fe_2O_3 MC + ZnO NR mixture in water. Reaction conditions for (**b**) and (**c**): 16 hours under broad spectrum light, 1 mg/mL catalyst, 1 M H_2O_2, 1:1 by vol. mixture of CH and water. Error bars are a standard deviation calculated from at least three replicate runs.

Figure S7:X-ray diffraction of HP Catalysts including nanodisc functionalization and components.

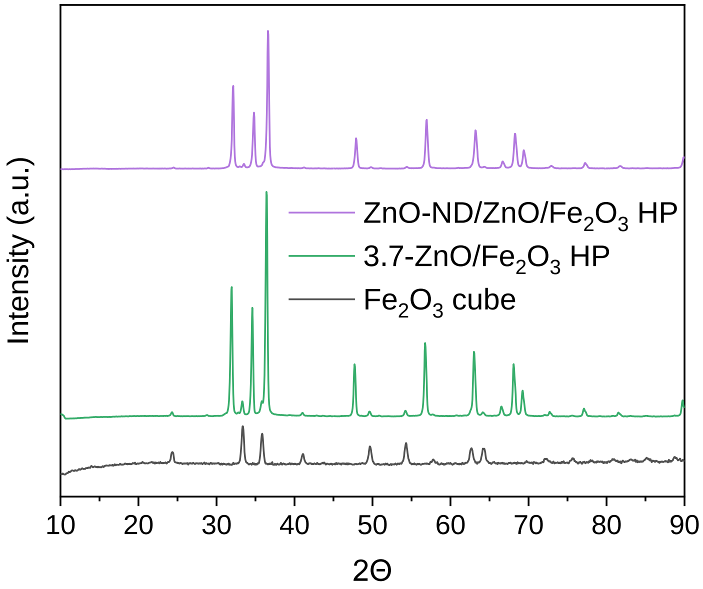

Fig. S7: X-ray diffraction of Fe_2O_3 cubes (scaled 5x), 3.7-ZnO/Fe_2O_3 HP, and ZnO-ND/ZnO/Fe_2O_3 HP.

ZnO-ND/ZnO/Fe_2O_3 HP were synthesized using an adapted literature procedure[12] to purposefully encapsulate ZnO spikes with concentric hexagonal nanodiscs. Briefly, 1 g 3.7-ZnO/Fe_2O_3 HPs were dispersed in 1.6 L DI water containing 25 mM zinc nitrate hexahydrate, 200 mM hexamethylene tetramine, and 0.2 mM sodium citrate tribasic dihydrate. The solution was heated to ca 90°C and sonicated using a ultrasonic pilot-scale Hieslcher UIP 1000HdT reactor for four hours. After sonication, the ND HPs were purified by allowing the ND HPs to settle and removing the excess reaction effluent and loose ZnO particles produced during synthesis. Purification continued until all excess ZnO was removed.

Figure S8: Epoxide selectivity of catalysts in cyclohexane oxidation.

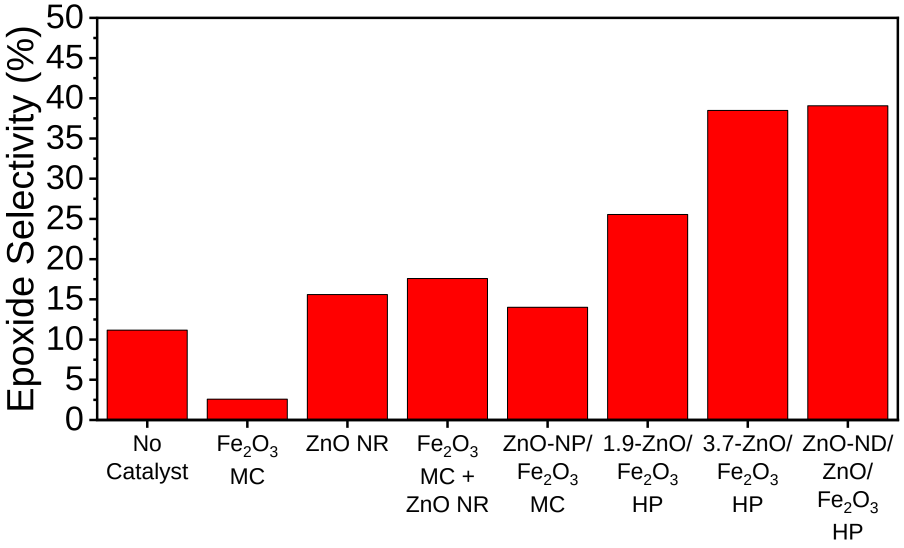

Fig. S8: Epoxide selectivity calculated for various catalysts under standard reaction conditions. Experimental conditions: 16 hours, 1 M H_2O_2, 1 mg/mL catalyst, 1:1 by vol. mixture of CH and water.

Figure S9: Product concentration as a function of HP catalyst concentration

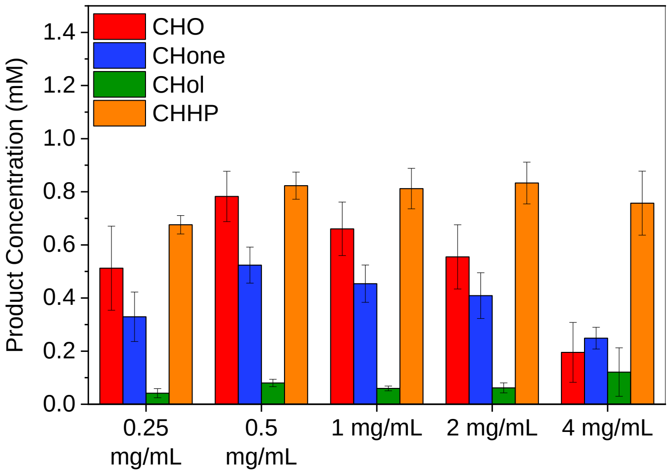

Fig. S9: Product concentration as a function of 3.7-ZnO/Fe_2O_3 HP catalyst concentration. *This dataset was collected at a different light intensity than other experiments in this work and is therefore only internally comparable within this dataset*. Experimental conditions: 16 hours, 1 M H_2O_2, 1:1 by vol. mixture of CH and water. Error bars are a standard deviation calculated from at least three replicate runs.

Figure S10: Photoluminescence emission spectra of microcube and nanorod mixture and non-normalized data from Figure 4d

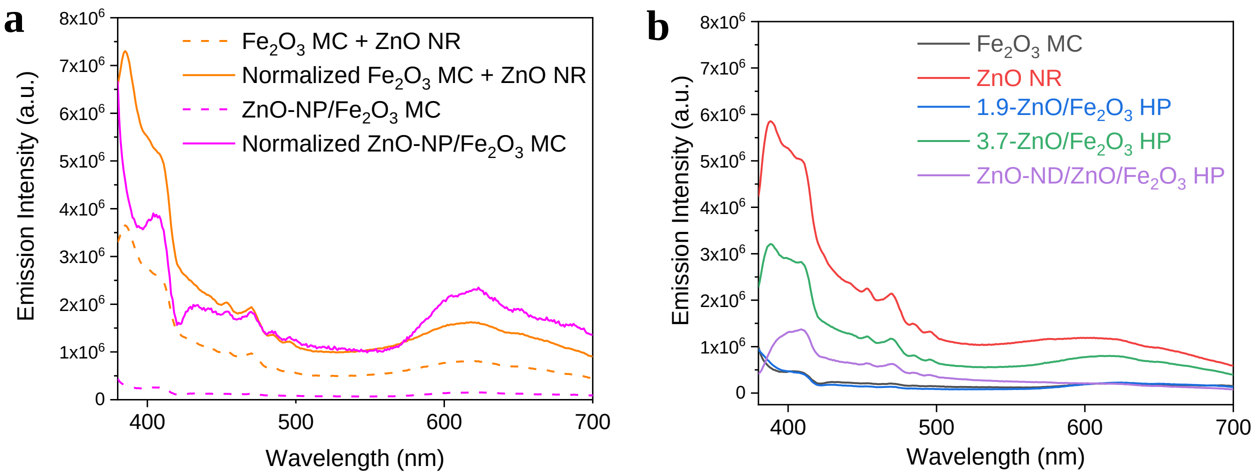

Fig. S10: Photoluminescence emission spectra from excitation at 360 nm of (**a**) heterogeneous 1:1 by mass mixture of Fe_2O_3 MC and ZnO NR (Fe_2O_3 MC + ZnO NR) and the ZnO-NP/Fe_2O_3 MC both with and without normalization by ZnO mass content and (**b**) photoluminescence emission spectra of catalysts shown in **Figure 4d** without normalization. ZnO-NP/Fe_2O_3 MC ZnO content estimated using average ZnO nanoparticle size and count of nanoparticles on the face of Fe_2O_3 MC.

The PL spectroscopy shown in **Fig. 4d** supports our experimental findings that larger spikes lead to higher product yields consistent with previous work[13] and a large reduction of recombination, except for the 1.9-ZnO/Fe_2O_3 HPs. These HPs had lower product yield but showed the lowest e^-/h^+ recombination. With these HPs, we suspect that hematite core can absorb light (yet still not contribute to the photocatalytic reaction) unlike HPs with thicker and longer nanorods, evidenced by its deep red color like hematite cubes (**Fig. 1**). With epoxide formation dependent on ZnO light absorption, we suspect the hematite core light absorption lowers the photon efficiency by hindering epoxide formation in this case. Additional PL spectroscopy is shown below in **Fig. S10**.

Table S3 Cyclohexene yield for select single experiments at low and high oxidant concentrations.

Reaction conditions: 16 hours, 1 mg/mL catalyst, 1:1 by vol. mixture of CH and aqueous H_2O_2. H_2O_2 concentration calculated over the total volume (30 mL).

Catalyst	Oxidant Concentration (M)	Cyclohexene Production (mM)
Fe_2O_3 MC	0.012	0.09
	1	0.15
ZnO NR	0.012	0.08
	1	0.09
3.7-ZnO/Fe_2O_3 HP	0.012	0.09
	1	0.16
ZnO-ND/ZnO/Fe_2O_3 HP	0.012	0.10
	1	0.23
Au-NP/ZnO/Fe_2O_3 HP	0.012	3.37
	1	0.64

Figure S11: Product yield of HP catalyst with designated concentration of products added to reaction flask

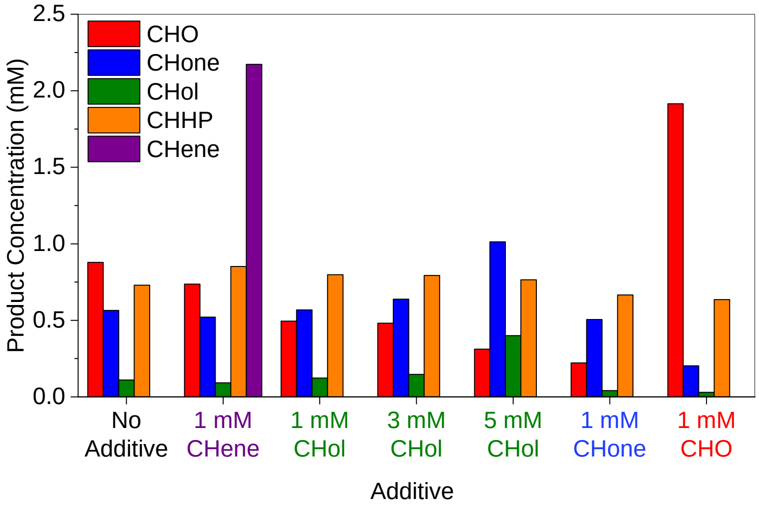

Fig. S11: Single experiment product yield with designated concentration of products added to reaction flask. Additive added at 1 mM for total volume (30 mL, water + CH), but only cyclohexane phase analyzed by GC (1 mM total = 2 mM in cyclohexane if no transfer to aqueous phase). Experimental conditions: 16 hours, 1 mg/mL 3.7-ZnO/Fe_2O_3 HP, 1 M H_2O_2, 1:1 by vol. mixture of CH and water.

Another potential pathway considered for epoxidation was the dehydration of CHol to CHene, which has been shown previously on Cu-ZnO catalysts and is dependent on the acidic sites on the catalytic surface.[9] CHol dehydrogenation to CHone is also widely reported.[9] Adding CHol to the reaction mixture did not lead to quantifiable CHene production and decreased CHO and CHone production compared to the results of **Fig. 3a**. At high CHol concentrations (5 mM), we see an increase in the selectivity and overall yield of CHone compared to **Fig. 3a**, indicating that CHol can be further oxidized to CHone at those conditions.[9] However, at lower cyclohexanol concentrations (3 mM, 1 mM), there is no increase in CHone or CHO production but there is obvious consumption of the cyclohexanol added, indicating that cyclohexanol is likely fully oxidized to CO_2 at our reaction conditions rather than dehydrated to CHene (**Fig. S11**).[10,11]

As seen in **Fig. S11**, CHone and CHO were also both added to the reaction mixture. CHone is also consumed in the reaction and inhibits the production of CHO, while added CHO is not consumed but does inhibit the production of CHone. This further indicates competition of these species, possibly for adsorption sites on the catalyst surface.

Figure S12: TEM images and UV-Vis extinction spectra of Au-NP/ZnO/Fe_2O_3 HP

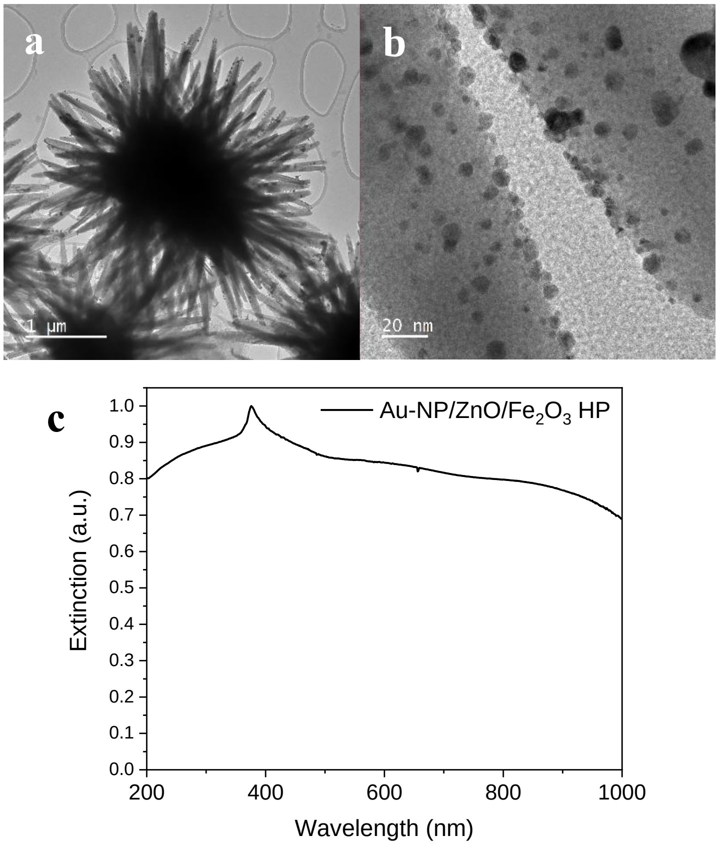

Fig. S12: (**a**, **b**) TEM images of Au-NP/ZnO/Fe_2O_3 HP at different magnifications and (**c**) normalized UV-Vis extinction spectra. TEM images were collected on a Thermo Fisher Talos F200X G2 S/TEM.

Au-NP/ZnO/Fe_2O_3 HPs were synthesized using deposition-precipitation of gold chloride with urea.[14] 500 mg of 3.7-ZnO/Fe_2O_3 HP were dispersed in 50 mL of ultrapure water containing 4.2 mM gold chloride trihydrate and 0.42 M urea. The mixture was stirred at 80°C under reflux in the dark for 16 hours. The particles were then centrifuged and washed with water 3x at 1000 RPM to remove excess precursor. The particles were then lyophilized and calcined at 400°C for 4 hours in air. Finally, the particles were washed with water 3x and lyophilized again. Gold was confirmed using EDX. **Fig. S12** contains TEM images and additional spectroscopic characterization of the Au-NP/ZnO/Fe_2O_3 HP.

Figure S13: Cyclohexane oxidation results with recycled HP Catalyst

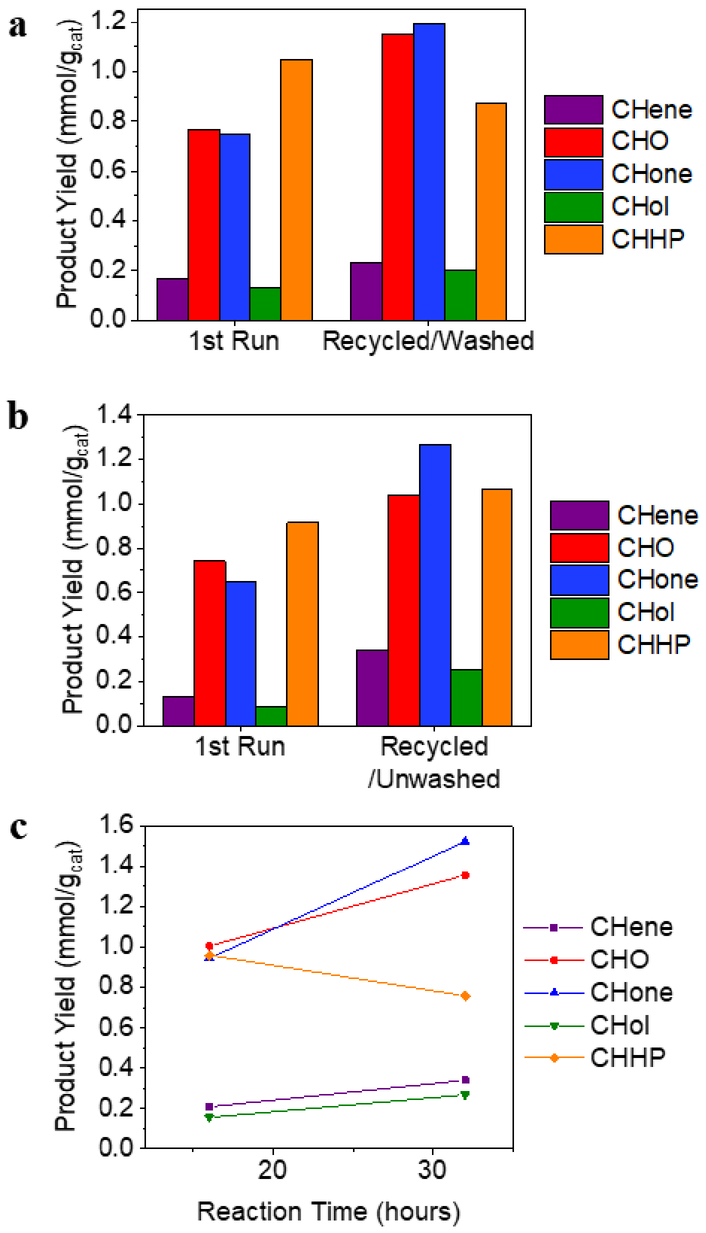

Fig. S13: Results from reactions where 3.3-ZnO/Fe_2O_3 HP was recycled. (a) Emulsion was broken, the biphasic supernatant was removed, and the catalyst was washed with ethanol and water and dried for 3 hours at 70°C, (b) emulsion was broken, supernatant removed, and catalyst was dried in residual supernatant for 3 hours at 70°C, and (c) HP was left in original reagents and was shaken vigorously to reform the emulsion and the reaction was restarted. Reaction conditions: 1 mg/mL 3.3-ZnO/Fe_2O_3 HP in a 1:1 by vol emulsion of CH and aqueous 1 M H_2O_2.

Figure S14: XPS spectra and SEM of recycled HP catalyst

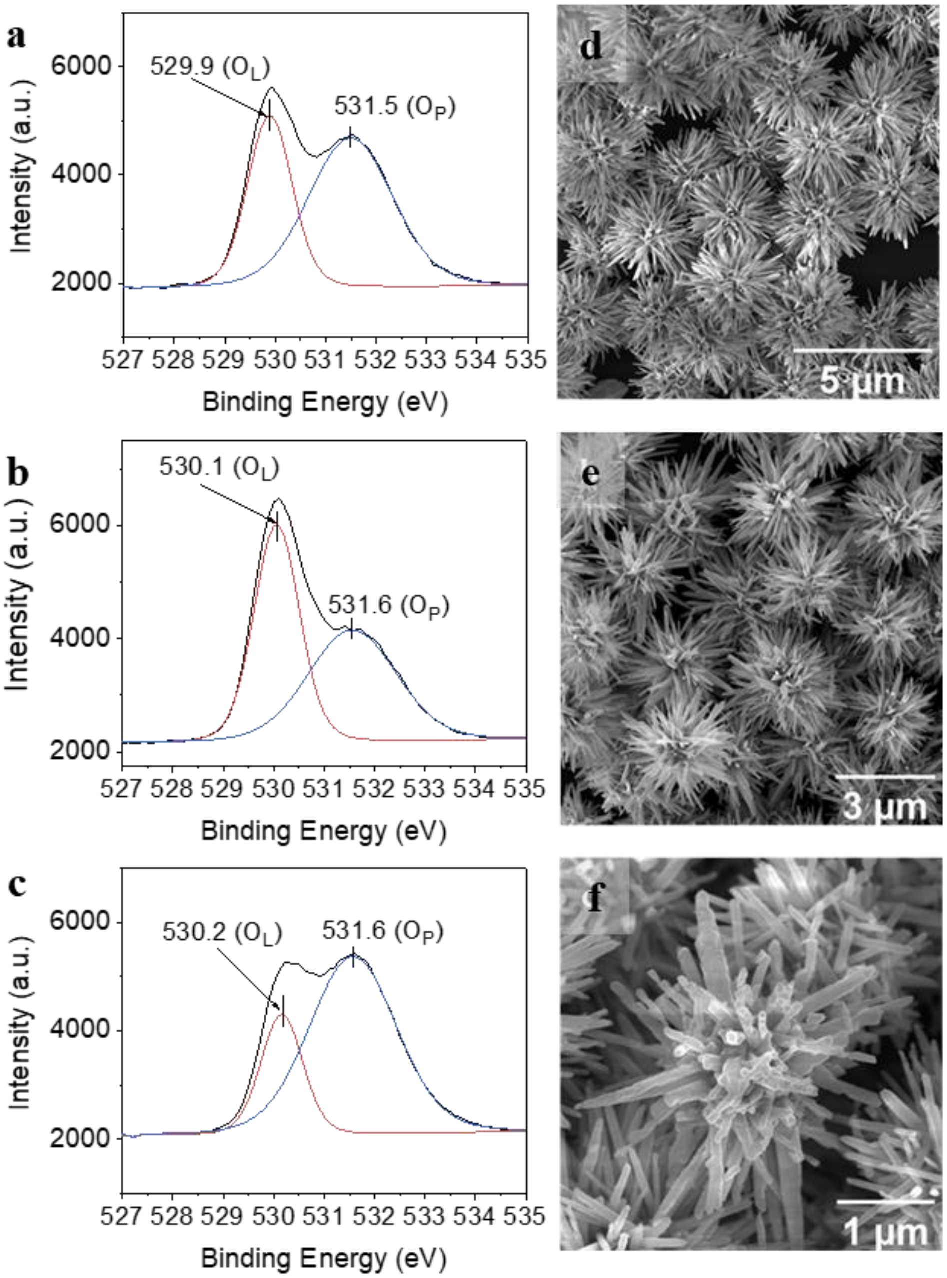

Fig. S14: O1s XPS spectra (a,b,c) and corresponding SEM image for (a,d) fresh 3.3-ZnO/Fe_2O_3 HP, (b,e) spent 3.3-ZnO/Fe_2O_3 HP after reaction without added H_2O_2, (c,f) spent 3.3-ZnO/Fe_2O_3 HP after reaction with 1 M H_2O_2. Reaction conditions: 1 mg/mL 3.3-ZnO/Fe_2O_3 HP in a 1:1 by vol emulsion of CH and water or aqueous H_2O_2 for 16 hours under broad spectrum light.

Figure S15: Light intensity for X-Cite Series 120 lamp used in cyclohexane oxidation experiments

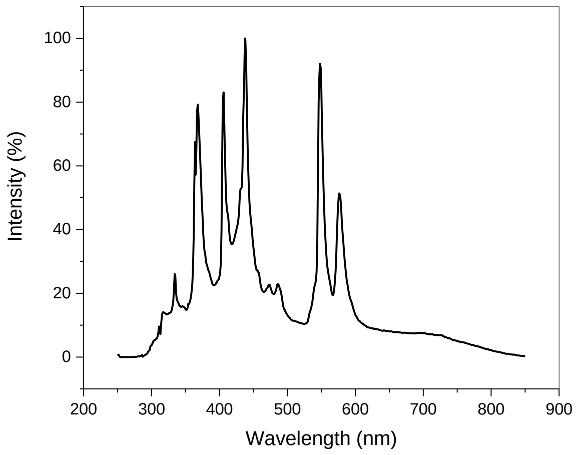

Fig. S15: Light intensity for X-Cite Series 120 lamp used for all experiments in this work.

Figure S16: Cyclohexane oxidation product distribution from various light and heat condition

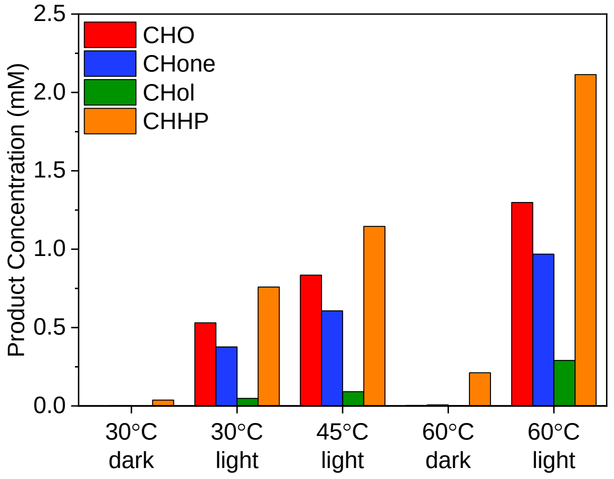

Fig. S16: Single-experiment product distribution from the oxidation of cyclohexane in a 1:1 (by vol.) cyclohexane/aqueous 1M H_2O_2 emulsion with 1 mg/mL 3.7-ZnO/Fe_2O_3 HP with various heat and light conditions. *This dataset was collected at a different light intensity than other experiments in this work and is therefore only internally comparable.* All experiments in this figure are single runs.

Figure S17: GC-MS mass spectrum identification of cyclohexene oxide in solution after reaction

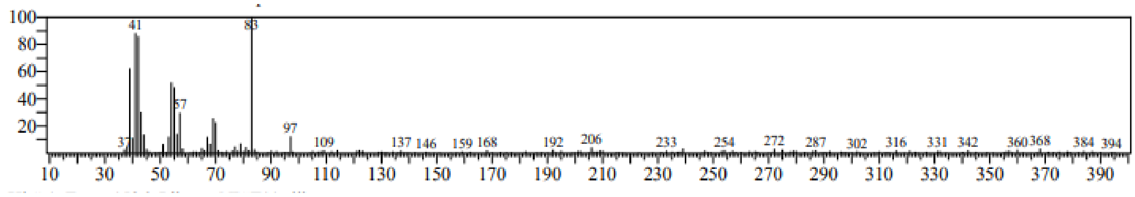

Fig. **S17**: GC-MS mass spectrum identification of cyclohexene oxide in solution after reaction with 1 M H_2O_2 and 1 mg/mL HP (SI=91). Cyclohexene oxide was also confirmed and quantified by running a series of calibration standards in GC-FID to confirm identical retention time to a product peak seen after reaction.

Supplementary References

1. Zhang, Z. *et al.* Electrospun nanofibers of p-type NiO/n-type ZnO heterojunctions with enhanced photocatalytic activity. *ACS Applied Materials and Interfaces* **2**, 2915–2923 (2010).

2. Gao, M., Zhu, L., Ong, W. L., Wang, J. & Ho, G. W. Structural design of TiO_2-based photocatalyst for H_2 production and degradation applications. *Catalysis Science and Technology* **5**, 4703–4726 (2015).

3. Tama, A. M. *et al.* MoS_2 nanosheet incorporated α-Fe_2O_3/ZnO nanocomposite with enhanced photocatalytic dye degradation and hydrogen production ability. *RSC Advances* **9**, 40357–40367 (2019).

4. Han, H. *et al.* α-Fe_2O_3/TiO_2 3D hierarchical nanostructures for enhanced photoelectrochemical water splitting. *Nanoscale* **9**, 134–142 (2017).

5. Lu, Y. R. *et al.* Effect of Fe_2O_3 coating on ZnO nanowires in photoelectrochemical water splitting: A synchrotron x-ray spectroscopic and spectromicroscopic investigation. *Solar Energy Materials and Solar Cells* **209**, (2020).

6. Dodd, A., McKinley, A., Tsuzuki, T. & Saunders, M. Tailoring the photocatalytic activity of nanoparticulate zinc oxide by transition metal oxide doping. *Materials Chemistry and Physics* **114**, 382–386 (2009).

7. Abdul Hamid, H., Lockman, Z., Hattori, T. & Abdul Razak, K. Sensitive and selective chloroform sensor using Fe_2O_3 nanoparticle-decorated ZnO nanorods in an aqueous solution. *Journal of Materials Science: Materials in Electronics* **30**, 18990–19000 (2019).

8. Kirchner, S. R. *et al.* Scattering Properties of Individual Hedgehog Particles. *Journal of Physical Chemistry C* **122**, 12015–12021 (2018).

9. Ji, D., Zhu, W., Wang, Z. & Wang, G. Dehydrogenation of cyclohexanol on Cu-ZnO/SiO_2 catalysts: The role of copper species. *Catalysis Communications* **8**, 1891–1895 (2007).

10. Raja, R. & Ratnasamy, P. Oxidation of cyclohexane over copper phthalocyanines encapsulated in zeolites. *Catalysis Letters* **48**, 1–10 (1997).

11. Du, P., Moulijn, J. A. & Mul, G. Selective photo(catalytic)-oxidation of cyclohexane: Effect of wavelength and TiO_2 structure on product yields. *Journal of Catalysis* **238**, 342–352 (2006).

12. Kim, D. *et al.* Hierarchical assembly of ZnO nanowire trunks decorated with ZnO nanosheets for lithium ion battery anodes. *RSC Advances* **10**, 13655–13661 (2020).

13. Montjoy, D. G. *et al.* Photocatalytic Hedgehog Particles for High Ionic Strength Environments. *ACS Nano* **15**, 4226–4234 (2021).

14. Zanella, R., Delannoy, L. & Louis, C. Mechanism of deposition of gold precursors onto TiO_2 during the preparation by cation adsorption and deposition-precipitation with NaOH and urea. *Applied Catalysis A: General* **291**, 62–72 (2005).
